# Zonulin-Assessed Intestinal Permeability and Body Composition Changes in Systemic Sclerosis

**DOI:** 10.3390/jcm15145338

**Published:** 2026-07-08

**Authors:** Antonietta Gigante, Chiara Pellicano, Maria Ludovica Iacovino, Carmen Gallicchio, Alessandra Oliva, Edoardo Rosato, Maurizio Muscaritoli

**Affiliations:** 1 Department of Translational and Precision Medicine, Sapienza University of Rome, 00185 Rome, Italy; antonietta.gigante@uniroma1.it (A.G.); iacovino.1900591@studenti.uniroma1.it (M.L.I.); carmen.gallicchio@uniroma1.it (C.G.); edoardo.rosato@uniroma1.it (E.R.); maurizio.muscaritoli@uniroma1.it (M.M.); 2 Department of Public Health and Infectious Diseases, Sapienza University of Rome, 00185 Rome, Italy; alessandra.oliva@uniroma1.it

**Keywords:** systemic sclerosis, zonulin, intestinal permeability, bioelectrical impedance analysis, body composition, sarcopenia

## Abstract

**Background/Objectives**: Systemic sclerosis (SSc) is a chronic autoimmune disease characterized by endothelial dysfunction, immune system dysregulation, and fibrosis of the skin and internal organs. Gastrointestinal involvement occurs in over 90% of patients and contributes to metabolic alterations and changes in body composition. Given the lack of specific markers of organ damage, zonulin has been investigated as a potential indicator of intestinal permeability. This study aims to evaluate the relationship between intestinal permeability, assessed by zonulin levels, and changes in body composition in SSc patients. **Methods**: Forty patients meeting EULAR criteria were included. Clinical evaluation, biochemical assessment including zonulin, and body composition analysis using bioelectrical impedance analysis (BIA) were performed. The BIA assessed the following parameters: fat-free mass index (FFMI; kg/m^2^); body cellular mass index (BCMI); fat mass index (FMI; kg/m^2^) and visceral fat area (VFA; cm^2^). **Results**: 32 of 40 patients enrolled were female (80%) with a median age of 55 years (IQR 49–62.5). Median zonulin level was 0.92 pg/mL (IQR 0.86–1.05). Reduced FFMI was present in 35% of patients and was associated with higher zonulin levels [1.16 pg/mL (IQR 0.91–1.34) vs. 0.92 pg/mL (IQR 0.83–0.96), *p* < 0.01]. Zonulin correlated negatively with BCMI (r = −0.332, *p* < 0.05), FFM (r = −0.302, *p* < 0.05) and muscle mass (r = −0.276, *p* < 0.05). **Conclusions**: Elevated serum zonulin levels, reflecting increased intestinal permeability, are associated with adverse body composition changes in SSc, suggesting gut barrier dysfunction as a potential mechanism of muscle loss and a therapeutic target in SSc.

## 1. Introduction

Systemic sclerosis (SSc) is a chronic autoimmune connective tissue disease characterized by immune dysregulation, progressive microvascular injury, and excessive extracellular matrix deposition, ultimately leading to fibrosis of the skin and multiple internal organs [1,2].

Among the organs affected, the gastrointestinal (GI) tract represents one of the most frequently involved systems, with clinical manifestations occurring in up to 90% of patients during the course of the disease. Gastrointestinal dysfunction may affect any segment of the digestive tract and commonly results in esophageal dysmotility, gastroesophageal reflux disease, delayed gastric emptying, small intestinal bacterial overgrowth (SIBO), and, in advanced cases, severe intestinal dysmotility or megacolon. SIBO has been reported in approximately 12–55% of patients with SSc and is associated with symptoms including abdominal bloating, distension, flatulence, and diarrhea, all of which may substantially impair quality of life and nutritional status [3].

Malnutrition and unfavorable body composition changes are increasingly acknowledged as clinically relevant manifestations of systemic sclerosis, particularly among patients with gastrointestinal involvement. Malnutrition and alterations in body composition arise through multiple interacting mechanisms, including reduced oral intake, impaired gastrointestinal motility, malabsorption of nutrients, increased resting energy expenditure, and reduced physical activity secondary to musculoskeletal and cutaneous involvement [4,5].

These factors frequently lead to a progressive reduction in fat-free mass (FFM), body cell mass (BCM), and skeletal muscle mass, all of which have been associated with worse functional status and poorer clinical outcomes [6].

Growing evidence suggests that intestinal barrier dysfunction may represent an additional mechanism contributing to these metabolic abnormalities. Zonulin, a physiological modulator of epithelial tight junctions, plays a key role in regulating intestinal permeability. Increased zonulin release promotes disruption of tight junction integrity, facilitating the passage of luminal microorganisms, toxins, and antigens into the systemic circulation. This process may amplify immune activation and promote chronic low-grade inflammation through sustained cytokine production [7]. Although GI involvement and nutritional impairment have been extensively described in SSc, the potential relationship between intestinal permeability and body composition has received little attention. In particular, whether circulating zonulin levels reflect alterations in muscle and fat compartments has not yet been systematically investigated.

Therefore, the aim of the present study was to evaluate the association between serum zonulin concentrations, as a marker of intestinal barrier dysfunction, and body composition assessed by bioelectrical impedance analysis (BIA) in SSc patients.

## 2. Materials and Methods

### 2.1. Study Population

This single-center observational study enrolled 40 consecutive SSc patients diagnosed according to the 2013 European League Against Rheumatism (EULAR) classification criteria [8]. At study entry, all participants underwent a comprehensive clinical evaluation, laboratory testing including serum zonulin, neutrophil-to-lymphocyte ratio (NLR), platelet-to-lymphocyte ratio (PLR), and BIA. Patients were excluded if they had conditions potentially affecting intestinal permeability or body composition, including eating disorders, malignancies, overlap connective tissue diseases, neurodegenerative disorders, acute or chronic infections, chronic respiratory diseases, renal or hepatic insufficiency, thyroid disorders, or clinically relevant cardiovascular diseases. At enrollment, all patients were receiving calcium channel blockers (nifedipine 20–30 mg/day), whereas none had been treated with immunosuppressive drugs or corticosteroids at a prednisone-equivalent dose of ≥10 mg/day. All patients were receiving proton pump inhibitors (PPIs) at the time of enrollment. The clinical evaluation of patients included the following parameters: skin involvement, distinguishing the study population into limited cutaneous (lc) and diffuse cutaneous (dc) forms of the disease; the degree of skin thickening, assessed using modified Rodnan skin score (mRSS); the disease severity scale (DSS), estimated by evaluating general health, peripheral blood vessels, skin, joints/tendons, muscles, GI involvement, lungs with evaluation of Forced Vital Capacity (FVC), heart, and kidney involvement [9,10,11].

The study protocol complied with the principles of the Declaration of Helsinki and was approved by the Ethics Committee of Sapienza University (approval No. 0304). Written informed consent was obtained from all participants before enrollment.

### 2.2. Anthropometric Data and Body Composition

Body mass index (BMI) was calculated as weight (kg) divided by the square of height (m^2^) and expressed as kg/m^2^.

Body composition was assessed in all participants by bioelectrical impedance analysis (BIA) using a multi-frequency body composition analyzer (InBody 770, InBody, Novate Milanese (MI), Italy), which delivers an alternating electrical current of 800 μA at a frequency of 50 Hz. All measurements were obtained in the morning after an overnight fast under standardized conditions. The following body composition parameters were evaluated: body cell mass index (BCMI), fat mass index (FMI, kg/m^2^), fat-free mass index (FFMI, kg/m^2^), visceral fat area (VFA, cm^2^), and intracellular and extracellular water. Gender-specific cut-off values for low FFMI were <15 kg/m^2^ in women and <17 kg/m^2^ in men, consistent with previous reports [12,13].

Phase angle (PhA) was calculated using the tetrapolar bioelectrical impedance method according to the following formula: PhA = (reactance × 180°)/(resistance × π). Based on previously published evidence, a cut-off value of 4.5° was used to define a low PhA.

### 2.3. Laboratory Assessment

Venous blood samples were collected under fasting conditions. Serum was separated by centrifugation at 2000 rpm for 10 min and stored at −80 °C until analysis.

Serum zonulin concentrations were determined in undiluted samples using a commercially available enzyme-linked immunosorbent assay (ELISA; Elabscience, Houston, TX, USA), following the manufacturer’s protocol, as previously reported [7].

Laboratory investigators performing the analyses remained blinded to all clinical and instrumental data throughout the study.

### 2.4. Statistical Analysis

Statistical analyses were conducted using IBM SPSS Statistics version 25.0 (IBM Corp., Armonk, NY, USA). Data distribution was assessed before statistical testing. Continuous variables are presented as median and interquartile range (IQR), whereas categorical variables are expressed as frequencies and percentages. Between-group comparisons were performed using Student’s t test or the Mann–Whitney U test, according to data distribution. Bonferroni adjustment was applied whenever multiple comparisons were performed. Categorical variables were compared using the chi-square test or Fisher’s exact test, as appropriate. Correlations between continuous variables were evaluated using Pearson’s or Spearman’s correlation coefficients depending on data distribution. Variables significantly associated with reduced FFMI in univariate analyses were subsequently entered into a multivariable logistic regression model to identify independent predictors. To minimize the risk of model overfitting, the number of covariates included in the multivariable analysis was limited according to the sample size and the number of outcome events. Statistical significance was defined as a two-sided *p* value < 0.05.

## 3. Results

The study cohort consisted of 32 females (80%), with a median age of 55 years (IQR 49–62.5) and a median BMI of 23.25 kg/m^2^ (IQR 21.35–24.96). Twenty-five patients (62.5%) had dcSSc and fifteen (37.5%) had lcSSc, with a median mRSS of 15.5 (IQR 9–22.5). The median serum zonulin was 0.92 pg/mL (IQR 0.86–1.05). The demographic and clinical characteristics of the study population are presented in Table 1.

The median FFMI was 16.4 kg/m^2^ (IQR 14.85–17.65) and 14 (35%) SSc patients had a reduced FFMI. Body composition parameters of SSc patients are summarized in Table 2.

**Figure 1:**
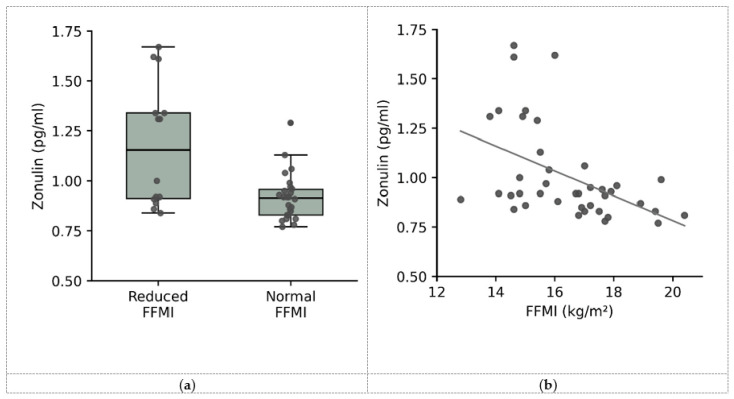
Relationship between serum zonulin and nutritional status assessed by fat-free mass index (FFMI). (**a**) Box plot shows comparative analysis of median serum zonulin levels between SSc patients with reduced versus normal FFMI (*p* = 0.008); (**b**) negative linear correlation between serum zonulin and FFMI in SSc patients (r= −0.486, *p* = 0.001).

SSc patients with reduced FFMI had statistically significantly higher serum zonulin [1.16 pg/mL (IQR 0.91–1.34) vs. 0.92 pg/mL (IQR 0.83–0.96), *p* = 0.008] (Figure 1a). SSc patients with reduced FFMI exhibited significant higher NLR [3.36 (IQR 2.66; 3.78) vs. 2.05 (IQR 1.85; 2.92), *p* = 0.015] and PLR [165.89 (IQR 130.73; 230.73) vs. 128.82 (IQR 91.87; 171.26), *p* = 0.045] compared to SSc patients with normal FFMI. Moreover, SSc patients with reduced FFMI had statistically significantly lower FVC [82.50% (IQR 74; 103) vs. 105.5% (IQR 90.5; 113.5), *p* = 0.016] than SSc patients with normal FFMI. These results are summarized in Table 3. Furthermore, serum zonulin levels showed a significant inverse correlation with FFMI (r = −0.486, *p* = 0.001) (Figure 1b).

**Figure 2:**
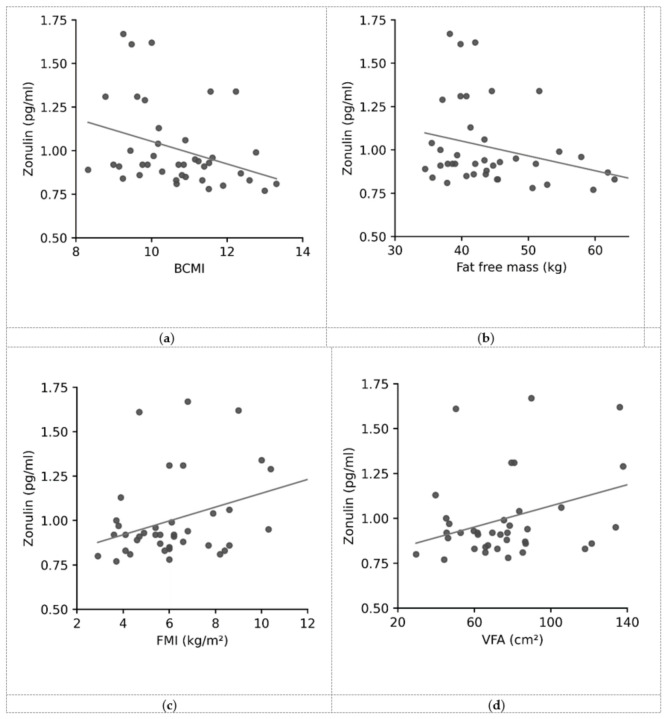
Relationship between serum zonulin and nutritional/body composition parameters. (**a**) Negative linear correlation between zonulin and Body Cellular Mass Index (BCMI) (r = −0.332, *p* = 0.018); (**b**) negative linear correlation between zonulin and fat-free mass (r = −0.302, *p* = 0.029); (**c**) positive linear correlation between zonulin and fat mass index (FMI) (r = 0.315, *p* = 0.024); (**d**) positive linear correlation between zonulin and Visceral Fat Area (VFA) (r = 0.317, *p* = 0.023).

Serum zonulin levels also showed a significant inverse correlation with both BCMI (r = −0.332, *p* = 0.018) and fat-free mass (r = −0.302, *p* = 0.029) (Figure 2a,b). In contrast, serum zonulin levels were positively correlated with both FMI (r = 0.315, *p* = 0.024) and VFA (r = 0.317, *p* = 0.023) (Figure 2c,d).

Finally, serum zonulin levels showed weak but statistically significant inverse correlations with total body water (r = −0.282, *p* = 0.039), intracellular water (r = −0.277, *p* = 0.042), extracellular water (r = −0.300, *p* = 0.030), body cell mass (r = −0.281, *p* = 0.040), and skeletal muscle mass (r = −0.276, *p* = 0.042).

Multivariate regression analysis showed that serum zonulin [B 10.07 S.E. 3.863 *p* = 0.009], and FVC [B −0.087 S.E. 0.033 *p* = 0.009] were independent predictors of reduced FFMI, with an accuracy of 81% (Table 4).

## 4. Discussion

The present study provides novel evidence supporting an association between intestinal barrier dysfunction and body composition abnormalities in patients with systemic sclerosis. Specifically, higher serum zonulin concentrations were associated with unfavorable body composition profiles, including lower fat-free mass, body cell mass, skeletal muscle mass, and body water compartments, together with higher fat mass and visceral adiposity. Furthermore, serum zonulin emerged as an independent predictor of reduced FFMI, alongside FVC, suggesting that intestinal permeability may represent an additional factor contributing to nutritional impairment in SSc.

Although gastrointestinal involvement is recognized as one of the hallmarks of systemic sclerosis, only limited evidence is currently available regarding the contribution of intestinal barrier dysfunction to nutritional status. Previous work from our group demonstrated that circulating zonulin levels are increased in patients with more severe gastrointestinal involvement, supporting its role as a surrogate marker of impaired intestinal barrier integrity [7]. The present findings expand those observations by showing that elevated serum zonulin is associated not only with gastrointestinal manifestations but also with multiple indicators of altered body composition, suggesting a broader systemic impact of gut barrier dysfunction.

Body composition abnormalities are increasingly recognized as clinically relevant features of SSc. The recent systematic review and meta-analysis by Radic et al. reported changes in body composition among patients with SSc, evaluated using BIA and DEXA [14]. The study confirmed that patients with SSc frequently exhibit reductions in fat-free mass, body cell mass, and phase angle, reflecting impaired nutritional status and progressive muscle loss [14]. This meta-analysis also highlighted that GI involvement (assessed using the University of California Los Angeles Scleroderma Clinical Trial Consortium Gastrointestinal Tract Instrument—UCLA questionnaire) and subsequent malnutrition are associated with, and likely contribute to, changes in body composition [14]. Gastrointestinal involvement has consistently been identified as one of the major determinants of malnutrition in this population. In the literature, the reported prevalence of malnutrition in SSc ranges from 6% to over 50% [15,16,17,18]. Differences in the reported prevalence of malnutrition across studies probably reflect the heterogeneity of the diagnostic criteria and nutritional assessment tools adopted rather than true differences between patient populations [15,16,17,18].

Nevertheless, across these studies, a reduction in muscle mass—affecting approximately 20–22% of patients—is consistently reported. Furthermore, in the study by Spanjer et al., data obtained from BIA were compared with those from DEXA, leading to the conclusion that BIA is a reliable tool for assessing body composition in patients with SSc [10,12].

Consistent with these observations, more than one-third of the patients included in our cohort had reduced FFMI. Importantly, these individuals exhibited significantly higher serum zonulin concentrations than patients with preserved muscle mass, while serum zonulin levels showed a moderate inverse correlation with FFMI. Taken together, these findings support the hypothesis that increased intestinal permeability may contribute to the development of unfavorable body composition changes in SSc.

Several biological mechanisms may explain this association. Gastrointestinal dysmotility in SSc frequently leads to fat and vitamin malabsorption, accompanied by excessive gas production and the accumulation of osmotically active substances, resulting in symptoms such as diarrhea, abdominal pain, and bloating. Delayed gastric emptying and prolonged orocecal transit promote SIBO, which is characterized by both an increased bacterial load and an altered microbial composition, with bacterial counts reported to be up to 60% higher than those observed in healthy individuals. Bloating and abdominal distension associated with dysbiosis may contribute to abdominal pain, leading to reduced appetite and decreased nutritional intake, thereby favoring the development of muscle wasting. Consistent with this hypothesis, previous studies have demonstrated an association between abdominal distension/bloating, assessed using the UCLA gastrointestinal score, and reduced FFMI in SSc patients [5].

Growing interest has recently focused on the concept of a “gut-muscle axis”, according to which alterations in gut microbiota composition and intestinal permeability may contribute to the age-related declines in muscle mass and function, directly influencing skeletal muscle metabolism. Experimental and clinical evidence suggests that disruption of intestinal barrier integrity promotes systemic exposure to bacterial endotoxins and inflammatory mediators, thereby favoring anabolic resistance, muscle protein breakdown, and progressive sarcopenia [19,20].

Although systemic inflammation is a well-established contributor to muscle wasting in both acute conditions (e.g., critically ill patients) and chronic diseases (e.g., cancer), its role in SSc appears to be less pronounced. Owing to the cross-sectional design of the present study, we cannot exclude the possibility that inflammatory activity was greater during the earlier stages of the disease. Nevertheless, previous findings from our group indicate that systemic inflammation in SSc is generally less prominent than would be expected for an autoimmune disease, particularly in patients with long-standing disease (median disease duration: 10 years) [21]. In our cohort, in fact, classical systemic inflammatory markers such as CRP were not significantly correlated with FFMI. These findings are consistent with the notion that CRP may not always be elevated in SSc, given its chronic, low-grade inflammatory profile, although increased CRP levels have been associated with disease activity and organ involvement in a subset of patients [22].

Conversely, the NLR, an inexpensive and widely available marker of systemic inflammation, was significantly associated with FFMI. Neutrophil-to-lymphocyte ratio values have been shown to be higher in SSc patients than in controls and to correlate with disease severity and specific complications such as interstitial lung disease and pulmonary arterial hypertension, suggesting that even low-grade systemic inflammation may contribute to muscle wasting in SSc [23].

Our study also shows a positive linear correlation between zonulin and FMI or VFA, which indicate fat mass and visceral fat, respectively.

Similar findings have been reported in individuals with metabolic disorders, in whom elevated serum zonulin levels have been associated with measures of central adiposity, including waist circumference and visceral adipose tissue [24,25,26,27,28,29,30,31,32]. Although the mechanisms underlying this association remain to be fully elucidated, these observations support a possible relationship between intestinal barrier dysfunction and alterations in body composition. Furthermore, serum zonulin was negatively correlated with total, intracellular, and extracellular body water, which may reflect impaired nutritional status in patients with SSc. Overall, our findings suggest that increased intestinal permeability is associated with unfavorable body composition parameters in SSc. However, longitudinal studies are needed to determine the clinical significance of serum zonulin and to clarify whether it may represent a useful biomarker of nutritional status and body composition changes in this population.

Our findings may also have potential clinical implications. The observed association between serum zonulin and body composition parameters suggests that intestinal barrier dysfunction could contribute to the nutritional impairment frequently observed in patients with SSc. However, given the cross-sectional design of the present study, these findings should be considered hypothesis-generating, and further longitudinal studies are needed to clarify the clinical significance of serum zonulin in this setting. Evidence from chronic respiratory diseases, particularly chronic obstructive pulmonary disease (COPD), has shown that elevated serum zonulin levels are associated with reduced FFM and poorer clinical outcomes, supporting a possible relationship between intestinal barrier dysfunction and muscle wasting [33,34,35,36]. Although SSc is characterized by a restrictive rather than an obstructive pulmonary phenotype, our finding that reduced FVC independently predicted low FFMI suggests that respiratory impairment and nutritional status may also be closely interconnected in this disease [37]. Future prospective studies should investigate whether nutritional interventions or strategies targeting intestinal dysbiosis and intestinal barrier dysfunction may improve nutritional status and body composition in patients with SSc.

From a clinical perspective, our findings suggest that serum zonulin may represent an early biomarker of nutritional impairment in patients with SSc. Although causality cannot be inferred from this cross-sectional analysis, the observed associations provide a rationale for future longitudinal studies investigating whether interventions targeting intestinal permeability or gut microbiota composition may preserve muscle mass and improve nutritional status in this population.

The present study has several limitations. First, the relatively small sample size reflects the rarity of SSc and may have limited the statistical power of some analyses. Second, the single-center, cross-sectional design precludes any inference of causality between serum zonulin levels and body composition changes. Furthermore, treatment adherence was not specifically evaluated and may have influenced the observed associations.

Despite these limitations, our findings provide novel evidence of an association between serum zonulin levels and body composition parameters in patients with SSc. These results support further prospective studies to better define the role of intestinal barrier dysfunction in nutritional impairment and to evaluate whether interventions targeting the gut may improve body composition and clinical outcomes in this population.

## 5. Conclusions

In conclusion, serum zonulin levels were significantly associated with body composition parameters in patients with systemic sclerosis, particularly with reduced FFM. These findings suggest that intestinal barrier dysfunction may contribute to nutritional impairment in SSc. Nevertheless, because of the cross-sectional nature of the study, these results should be interpreted as hypothesis-generating. Larger prospective studies are needed to confirm these associations, clarify the underlying mechanisms, and determine the clinical utility of serum zonulin as a marker of intestinal barrier dysfunction and nutritional status in patients with SSc.

## Figures and Tables

**Table 1:** Demographic and clinical characteristics of systemic sclerosis (SSc) patients enrolled (*n* = 40).

Variables	
Age, years, median and IQR	55 (49; 62.5)
Female, *n* (%)	32 (80)
dcSSc, *n* (%)	25 (62.5)
Early, *n* (%)	5 (12.5)
Active, *n* (%)	10 (25)
Late, *n* (%)	25 (62.5)
Anti-topoisomerase I, *n* (%)	19 (47.5)
Anti-centromere, *n* (%)	10 (25)
Anti-RNA polymerase III, *n* (%)	4 (10)
Duration of disease, years, median and IQR	13 (8; 20)
mRSS, median and IQR	15.5 (9; 22.5)
DAI, median and IQR	3.22 (1.43; 4)
DSS, median and IQR	7 (4; 9)
ESR, mm/h, median and IQR	23 (11.5; 31.25)
CRP, µg/L, median and IQR	1500 (900; 3325)
Zonulin, pg/mL, median and IQR	0.92 (0.86; 1.05)

BMI, Body Mass Index; dcSSc, diffuse cutaneous Systemic Sclerosis; mRSS, modified Rodnan Scleroderma Score; DAI, Disease Activity Index; DSS, Disease Severity Score; ESR, Erythrocyte Sedimentation Rate; CRP, C-reactive protein.

**Table 2:** Body composition of Systemic Sclerosis (SSc) patients enrolled (*n* = 40).

Variables	
BMI, kg/m^2^, median and IQR	23.25 (21.35; 24.96)
Fat free mass, kg, median and IQR	42.7 (38.8; 49.35)
Fat mass, kg, median and IQR	17.05 (12.8; 18.4)
Phase Angle (°), median and IQR	4.6 (4.05; 5.15)
Total water, L, median and IQR	31.3 (28.8; 36.05)
Intracellular water, L, median and IQR	19.25 (17.50; 22.30)
Extracellular water, L, median and IQR	12.2 (11.25; 13.8)
BCMI, kg/m^2^, median and IQR	10.69 (9.72; 11.52)
Cellular mass, kg, median and IQR	27.50 (25.05; 31.95)
Muscle mass, kg, median and IQR	23.20 (20.80; 27.10)
FMI, kg/m^2^, median and IQR	6 (4.65; 7.80)
FFMI, kg/m^2^, median and IQR	16.4 (14.85; 17.65)
Arm circumference, cm, median and IQR	28.8 (26.35; 30.1)
Waist/hips ratio, median and IQR	0.88 (0.83; 0.90)
VFA, cm^2^, median and IQR	76.15 (59.85; 87.25)

BMI, Body Mass Index; BCMI, Body Cellular Mass Index; FMI, Fat Mass Index; FFMI, Fat Free Mass Index; VFA, Visceral Fat Area.

**Table 3:** Univariate analyses of potential predictors of reduced fat-free mass index (FFMI) in systemic sclerosis (SSc) patients enrolled (*n* = 40).

Variables	Normal FFMI (*n* = 26)	Reduced FFMI (*n* = 14)	*p*-Value
Age, years, median (IQR)	57.0 (50.3–64.5)	54.0 (44.3–57.8)	0.334
Female sex, *n* (%)	19 (73.1)	13 (92.9)	0.222
Disease duration, years, median (IQR)	12.0 (8.8–19.0)	14.0 (8.3–27.5)	0.585
dcSSc, *n* (%)	14 (53.8)	11 (78.6)	0.177
Late NVC pattern, *n* (%)	14 (53.8)	11 (78.6)	0.177
Anti-topoisomerase I positivity, *n* (%)	14 (53.8)	9 (64.3)	0.739
ILD, *n* (%)	7 (26.9)	7 (50.0)	0.178
PAH, *n* (%)	1 (3.8)	1 (7.1)	1.000
mRSS, median (IQR)	12.0 (8.3–21.5)	18.5 (11.8–23.8)	0.164
DAI, median (IQR)	2.46 (0.78–3.81)	3.48 (2.38–4.92)	0.099
DSS, median (IQR)	7.0 (3.8–9.0)	7.5 (5.3–9.0)	0.455
ESR, mm/h, median (IQR)	15.5 (9.8–27.5)	26.0 (20.3–35.8)	0.096
CRP, µg/L, median (IQR)	1550 (975–2750)	1550 (650–8700)	0.927
DLCO, % predicted, median (IQR)	77.5 (70.3–85.5)	73.5 (56.8–82.3)	0.220
FVC, % predicted, median (IQR)	105.5 (90.5–113.5)	82.50 (74–103)	0.016
Serum zonulin, pg/mL, median (IQR)	0.92 (0.83–0.96)	1.16 (0.91–1.34)	0.008
NLR, median (IQR)	2.05 (1.85–2.92)	3.36 (2.66–3.78)	0.015
PLR, median (IQR)	128.82 (91.87–171.26)	165.89 (130.73–230.73)	0.045

FFMI, fat-free mass index; dcSSc, diffuse cutaneous systemic sclerosis; NVC, nailfold capillaroscopy; ILD, interstitial lung disease; PAH, pulmonary arterial hypertension; mRSS, modified Rodnan skin score; DAI, disease activity index; DSS, disease severity score; ESR, erythrocyte sedimentation rate; CRP, C-reactive protein; DLCO, diffuse capacity of the lung for carbon oxide; FVC, forced vital capacity; NLR, neutrophil-to-lymphocyte ratio; PLR, platelet-to-lymphocyte ratio.

**Table 4:** Multivariable logistic regression analysis with regression coefficient (B) and standard error (S.E.) for reduced fat-free mass index (FFMI).

Variables	B (S.E.)	*p*-Value
Zonulin	10.7 (3.863)	0.009
NLR	0.566 (0.306)	0.064
FVC	−0.087 (0.033)	0.009

NLR, neutrophil-to-lymphocyte ratio; FVC, Forced Vital Capacity.

## Data Availability

Data supporting the findings of this study are available from the corresponding author upon reasonable request.

## Abbreviations

The following abbreviations are used in this manuscript:

BCMI	Body Cellular Mass Index
BIA	Bioelectrical Impedance Analysis
BMI	Body Mass Index
COPD	Chronic Obstructive Pulmonary Disease
CRP	C-Reactive Protein
DAI	Disease Activity Index
dcSSc	Diffuse Cutaneous Systemic Sclerosis
DEXA	Dual-Energy X-ray Absorptiometry
DSS	Disease Severity Scale
ESR	Erythrocyte Sedimentation Rate
ELISA	Enzyme-Linked Immunosorbent Assay
EULAR	European League Against Rheumatism
FEV1	Forced Expiratory Volume in 1 s
FFM	Fat-Free Mass
FFMI	Fat-Free Mass Index
FMI	Fat Mass Index
FVC	Forced Vital Capacity
GIT	Gastrointestinal Tract
ID	Intestinal Damage
IQR	Interquartile Range
lcSSc	Limited Cutaneous Systemic Sclerosis
MT	Microbial Translocation
mRSS	Modified Rodnan Skin Score
NLR	Neutrophil-to-Lymphocyte Ratio
NVC	Nailfold capillaroscopy
PhA	Phase Angle
PLR	Platelet-to-Lymphocyte Ratio
SIBO	Small Intestinal Bacterial Overgrowth
SSc	Systemic Sclerosis
VAT	Visceral Adipose Tissue
VFA	Visceral Fat Area

## References

[1] Lepri G., Di Battista M., Codullo V., Bonomi F., Sulis A., Guiducci S., Della Rossa A. (2024). Systemic sclerosis: One year in review 2024. Clin. Exp. Rheumatol..

[2] Jerjen R., Nikpour M., Krieg T., Denton C.P., Saracino A.M. (2022). Systemic sclerosis in adults. Part I: Clinical features and pathogenesis. J. Am. Acad. Dermatol..

[3] Shreiner A.B., Murray C., Denton C., Khanna D. (2016). Gastrointestinal Manifestations of Systemic Sclerosis. J. Scleroderma Relat. Disord..

[4] Gigante A., Rosato E., Muscaritoli M. (2021). Body composition and microvascular damage in systemic sclerosis patients. J. Endocrinol. Investig..

[5] Rosato E., Gigante A., Pellicano C., Villa A., Iannazzo F., Alunni Fegatelli D., Muscaritoli M. (2022). Symptoms related to gastrointestinal tract involvement and low muscularity in systemic sclerosis. Clin. Rheumatol..

[6] Baptista G., Barazzoni R., Blaauw R., Coats A., Crivelli A., Evans D., Gramlich L., Fuchs-Tarlovsky V., Keller H., Llido L. (2019). GLIM criteria for the diagnosis of malnutrition—A consensus report from the global clinical nutrition community. Clin. Nutr..

[7] Pellicano C., Oliva A., Colalillo A., Gigante A., D’Aliesio E., Al Ismail D., Miele M.C., Cianci R., Mastroianni C.M., Rosato E. (2024). Serum markers of microbial translocation and intestinal damage in assessment of gastrointestinal tract involvement in systemic sclerosis. Clin. Exp. Med..

[8] van den Hoogen F., Khanna D., Fransen J., Johnson S.R., Baron M., Tyndall A., Matucci-Cerinic M., Naden R.P., Medsger T.A., Carreira P.E. (2013). 2013 classification criteria for systemic sclerosis: An American college of rheumatology/European league against rheumatism collaborative initiative. Ann. Rheum. Dis..

[9] LeRoy E.C., Black C., Fleischmajer R., Jablonska S., Krieg T., Medsger T.A., Rowell N., Wollheim F. (1988). Scleroderma (systemic sclerosis): Classification, subsets and pathogenesis. J. Rheumatol..

[10] Caimmi C., Caramaschi P., Venturini A., Bertoldo E., Vantaggiato E., Viapiana O., Ferrari M., Lippi G., Frulloni L., Rossini M. (2018). Malnutrition and sarcopenia in a large cohort of patients with systemic sclerosis. Clin. Rheumatol..

[11] Medsger T.A., Silman A.J., Steen V.D., Black C.M., Akesson A., Bacon P.A., Harris C.A., Jablonska S., Jayson M.I., Jimenez S.A. (1999). A disease severity scale for systemic sclerosis: Development and testing. J. Rheumatol..

[12] Spanjer M.J., Bultink I.E.M., de van der Schueren M.A.E., Voskuyl A.E. (2017). Prevalence of malnutrition and validation of bioelectrical impedance analysis for the assessment of body composition in patients with systemic sclerosis. Rheumatology.

[13] Clements P., Lachenbruch P., Siebold J., White B., Weiner S., Martin R., Weinstein A., Weisman M., Mayes M., Collier D. (1995). Inter and intraobserver variability of total skin thickness score (modified Rodnan TSS) in systemic sclerosis. J. Rheumatol..

[14] Radić M., Kolak E., Đogaš H., Gelemanović A., Bučan Nenadić D., Vučković M., Radić J. (2024). Body composition parameters in systemic sclerosis-a systematic review and meta-analysis. Rheumatology.

[15] Codullo V., Cereda E., Crepaldi G., Cappello S., Montecucco C., Caccialanza R., Caporali R. (2015). Disease-related malnutrition in systemic sclerosis: Evidences and implications. Clin. Exp. Rheumatol..

[16] Rosato E., Gigante A., Colalillo A., Pellicano C., Alunni Fegatelli D., Muscaritoli M. (2023). GLIM-diagnosed malnutrition predicts mortality and risk of hospitalization in systemic sclerosis: A retrospective study. Eur. J. Intern Med..

[17] Pardali E.C., Gkouvi A., Grammatikopoulou M.G., Mitropoulos A., Cholevas C., Poulimeneas D., Klonizakis M. (2025). Nutritional Status and Dietary Challenges in Patients with Systemic Sclerosis: A Comprehensive Review. Nutrients.

[18] Wojteczek A., Chmielewski M., Zdrojewski Z. (2024). Nutritional disorders and nutrition-related conditions: An underestimated clinical problem in systemic sclerosis. Reumatologia.

[19] Grosicki G.J., Fielding R.A., Lustgarten M.S. (2018). Gut Microbiota Contribute to Age- Related Changes in Skeletal Muscle Size, Composition, and Function: Biological Basis for a Gut-Muscle Axis. Calcif. Tissue Int..

[20] Ticinesi A., Nouvenne A., Cerundolo N., Catania P., Prati B., Tana C., Meschi T. (2019). Gut Microbiota, Muscle Mass and Function in Aging: A Focus on Physical Frailty and Sarcopenia. Nutrients.

[21] Tota Ł., Piotrowska A., Pałka T., Morawska M., Mikuľáková W., Mucha D., Żmuda-Pałka M., Pilch W. (2019). Muscle and intestinal damage in triathletes. PLoS ONE.

[22] Ross L., Stevens W., Rabusa C., Wilson M., Ferdowsi N., Walker J., Sahhar J., Ngian G.-S., Zochling J., Roddy J. (2018). The role of inflammatory markers in assessment of disease activity in systemic sclerosis. Clin. Exp. Rheumatol..

[23] Zinellu A., Mangoni A.A. (2024). The association between the neutrophil-to-lymphocyte ratio, platelet-to-lymphocyte ratio, and monocyte-to-lymphocyte ratio and systemic sclerosis and its complications: A systematic review and meta-analysis. Front. Immunol..

[24] Koutoukidis D.A., Yen S., Gomez Castro P., Misheva M., Jebb S.A., Aveyard P., Tomlinson J.W., Mozes F.E., Cobbold J.F., Johnson J.S. (2024). Changes in intestinal permeability and gut microbiota following diet-induced weight loss in patients with metabolic dysfunction-associated steatohepatitis and liver fibrosis. Gut Microbes.

[25] Bona M.D., Torres C.H.M., Lima S.C.V.C., Morais A.H.A., Lima A.Â.M., Maciel B.L.L. (2022). Intestinal Barrier Permeability in Obese Individuals with or without Metabolic Syndrome: A Systematic Review. Nutrients.

[26] Zak-Gołąb A., Kocełak P., Aptekorz M., Zientara M., Juszczyk L., Martirosian G., Chudek J., Olszanecka-Glinianowicz M. (2013). Gut microbiota, microinflammation, metabolic profile, and zonulin concentration in obese and normal weight subjects. Int. J. Endocrinol..

[27] Aasbrenn M., Lydersen S., Farup P.G. (2020). Changes in serum zonulin in individuals with morbid obesity after weight-loss interventions: A prospective cohort study. BMC Endocr. Disord..

[28] Stenman L.K., Lehtinen M.J., Meland N., Christensen J.E., Yeung N., Saarinen M.T., Courtney M., Burcelin R., Lähdeaho M.-L., Linros J. (2016). Probiotic with or Without Fiber Controls Body Fat Mass, Associated with Serum Zonulin, in Overweight and Obese Adults- Randomized Controlled Trial. EBioMedicine.

[29] Bonakdar R.A., Sweeney M., Dalhoumi S., Adair V., Garvey C., Hodge T., Herrala L., Barbee A., Case C., Kearney J. (2020). Detoxification Enhanced Lifestyle Intervention Targeting Endotoxemia (DELITE) in the Setting of Obesity and Pain: Results of a Pilot Group Intervention. Integr. Med..

[30] Pepe G., Corica D., Currò M., Aversa T., Alibrandi A., Ientile R., Caccamo D., Wasniewska M. (2024). Fasting and meal-related zonulin serum levels in a large cohort of obese children and adolescents. Front. Endocrinol..

[31] Küme T., Acar S., Tuhan H., Çatlı G., Anık A., Gürsoy Çalan Ö., Böber E., Abacı A. (2017). The Relationship between Serum Zonulin Level and Clinical and Laboratory Parameters of Childhood Obesity. J. Clin. Res. Pediatr. Endocrinol..

[32] Pacifico L., Bonci E., Marandola L., Romaggioli S., Bascetta S., Chiesa C. (2014). Increased circulating zonulin in children with biopsy-proven nonalcoholic fatty liver disease. World J. Gastroenterol..

[33] Bone A.E., Hepgul N., Kon S., Maddocks M. (2017). Sarcopenia and frailty in chronic respiratory disease. Chronic Respir. Dis..

[34] Mete B., Pehlivan E., Gülbaş G., Günen H. (2018). Prevalence of malnutrition in COPD and its relationship with the parameters related to disease severity. Int. J. Chronic Obstr. Pulm. Dis..

[35] Karim A., Muhammad T., Ustrana S., Qaisar R. (2021). Intestinal permeability marker zonulin as a predictor of sarcopenia in chronic obstructive pulmonary disease. Respir. Med..

[36] Qaisar R., Karim A., Muhammad T., Ahmad F., Marinho D.A., Arkadianos I., Alkahtani S. (2025). The Interface of a Leaky Gut with Reduced Sarcopenia-Related Quality of Life (SarQoL) in Patients with Chronic Obstructive Pulmonary Disease. Int. J. Chronic Obstr. Pulm. Dis..

[37] Cottin V., Brown K.K. (2019). Interstitial lung disease associated with systemic sclerosis (SSc-ILD). Respir. Res..

